# Bioinformatics analysis of LINC00426 expression in lung cancer and its correlation with patients' prognosis

**DOI:** 10.1111/1759-7714.13228

**Published:** 2019-11-05

**Authors:** Wenjun Du, Juan Sun, Jundong Gu, Shiwu Zhang, Tao Zhang

**Affiliations:** ^1^ Department of Spine Center Tianjin Union Medical Center (Tianjin People's Hospital) Tianjin China; ^2^ Department of Radiology Tianjin Union Medical Center (Tianjin People's Hospital) Tianjin China; ^3^ Department of Thoracic Surgery Tianjin Union Medical Center (Tianjin People's Hospital) Tianjin China; ^4^ Department of Pathology Tianjin Union Medical Center (Tianjin People's Hospital) Tianjin China; ^5^ Department of Trauma Tianjin Hospital Tianjin China

**Keywords:** Bioinformatics analysis, prognosis, LINC00426, long noncoding RNA, non‐small cell lung cancer

## Abstract

**Background:**

To investigate the expression of long noncoding RNA (lncRNA) LINC00426 (long intergenic nonprotein coding RNA 426) in non‐small cell lung cancer (NSCLC) patients and its correlation with their prognosis.

**Methods:**

The expression of long noncoding RNA LINC00426 of non‐small cell lung cancer (NSCLC) in The Cancer Genome Atlas (TCGA) database was screened. According to the expression level of LINC00426 in tumor tissue of NSCLC patients, the patients were divided into high and low LINC00426 expression groups. The correlation between LINC00426 expression group and the prognosis of the patient was analyzed by log‐rank test. A total of 72 NSCLC patients who had undergone surgery were retrospectively included in this study. LINC00426 relative expression of tumor and normal lung tissue of the included 72 NSCLC patients were examined by real‐time quantitative PCR assay. The correlation between LINC00426 expression and the patients’ clinical characteristics were also evaluated.

**Results:**

LINC00426 relative expression was not statistically different between cancer and normal tissue (*P* > 0.05) of NSCLC patients in the TCGA database. The amplification and deep deletion mutation of LINC00426 gene was found in 0.5% of NSCLC patients. The overall survival (OS) of the LINC00426 high expression group was significantly higher than that of the low expression group (HR = 0.81, *P* = 0.044), while there was no significant difference between the high and low expression group (HR = 0.97, *P* = 0.82) for disease‐free survival (DFS). LINC0042646 expression level was elevated in 46 cases in normal lung tissue compared to the tumor tissue of the 72 NSCLC patients. LINC0042646 expression level was significantly correlated with the clinical stage (*P* < 0.05).

**Conclusion:**

Long noncoding RNA LINC00426 was downregulated in the tumor tissue of NSCLC patients and correlated with poor prognosis.

## Introduction

Lung cancer is the most diagnosed malignant carcinoma of the respiratory system.^1^, ^2^ There are hundreds of thousands of new cases of lung cancer diagnosed worldwide every year. However, the exact mechanism of the occurrence and development of lung cancer has not yet been fully elucidated. In recent years, with the development of high‐throughput sequencing technology, more and more studies have proved that long‐chain noncoding RNA plays an important role in the occurrence and development of lung cancer and the biological function of invasion and metastasis.^3^, ^4^ Recent studies have shown that long noncoding RNA (lncRNA) plays an important role in dose compensation effect, epigenetic regulation, cell cycle regulation, cell differentiation, cell apoptosis and tumorigenesis.^5^, ^6^, ^7^ With the continuous development of high‐throughput transcriptome sequencing technology, more and more lncRNAs have been found, but the exact biological functions and mechanisms of most of these are not clear. LINC00426 is a newly discovered long noncoding RNA. Its expression level and biological function in lung cancer are at present unclear. Therefore, we analyzed the expression of LINC00462 in non‐small cell lung cancer and its relationship with prognosis using bioinformatics analysis.

## Methods

### LINC00426 expression analysis

The expression of LINC00426 in the TCGA database was screened with the limitation of human beings. LINC00426 gene relative expression between lung cancer tissues and normal lung tissues in NSCLC patients was compared in the condition of the LINC00426 gene relative expression difference more than twice (| Log2FC | > 1), *P* < 0.05.

### Survival analysis

The patients were divided into LINC00426 high and low expression groups according to the expression level of LINC00426 in the tumor tissue of NSCLC patients. A Kaplan‐Meier survival curve was drawn. The correlation between the LINC00426 expression and the patients’ prognosis was analyzed by log‐rank test.

### LINC00426 relative expression detected by q‐PCR

A total of 72 NSCLC patients who had undergone surgery were included in this retrospective study. All patients provided their written informed consent. The study was approved by the Medical Ethics Committee of Tianjin Union Medical Center. The research related to human use complied with all the relevant national regulations, institutional policies and was carried out in accordance with the tenets of the Helsinki Declaration, and approved by the Tianjin Union Medical Center Hospital's institutional review board. Tumor tissue and corresponding normal lung tissue of the 72 patients with lung cancer were lysed by Trizol. Total RNA was extracted via reverse transcription into cDNA. The relative expression of long noncoding RNA LINC00426 was detected by real‐time fluorescence quantitative PCR assay.

### Statistical analysis

The statistical analysis was performed using STATA11.0 statistical software ( http://www.stata.com ), the measurement data were expressed with $\bar{x}\pm s$ and comparison between groups was made based on the *t*‐test of the sample mean. The enumeration data were expressed with a relative number, and the comparison between groups was made based on the χ^2^ test. *P* < 0.05 was considered statistically different.

## Results

### LINC00426 expression in multiple tumors

**Figure 1:**
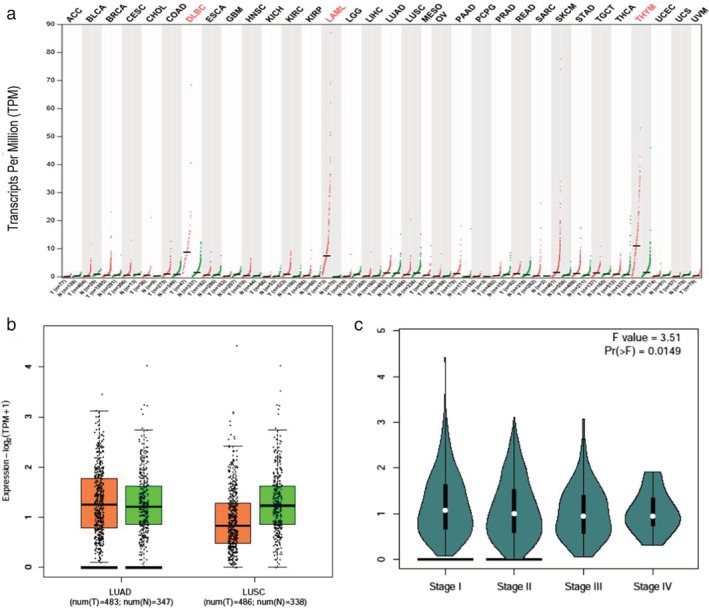
LINC00426 expression in tumor tissue and corresponding normal tissue (**a**) LINC00426 relative expression level in multiple tumors. (**b**) LINC00426 relative expression level in NSCLC between tumor tissue and corresponding normal tissue. (**c**) LINC00426 relative expression between different stages in NSCLC.

LINC00426 relative expression level was not statistically different between the tumor tissue and corresponding normal tissue in most of the malignant carcinomas, except for diffuse large B‐cell lymphoma (DLBCL), acute myeloid leukemia (AML), and thymoma (THYM) (Fig 1a). For lung cancer, the LINC00426 relative expression was not statistically different between cancer tissue and normal tissue (*P* > 0.05). LINC00426 expression level in lung squamous cell carcinoma was lower than that of tumor tissue (Fig 1b); however, the LINC00426 relative expression level was statistically different between the different clinical stages of lung cancer patients (*P* < 0.05) (Fig 1c).

### LINC00426 mutation status

**Figure 2:**
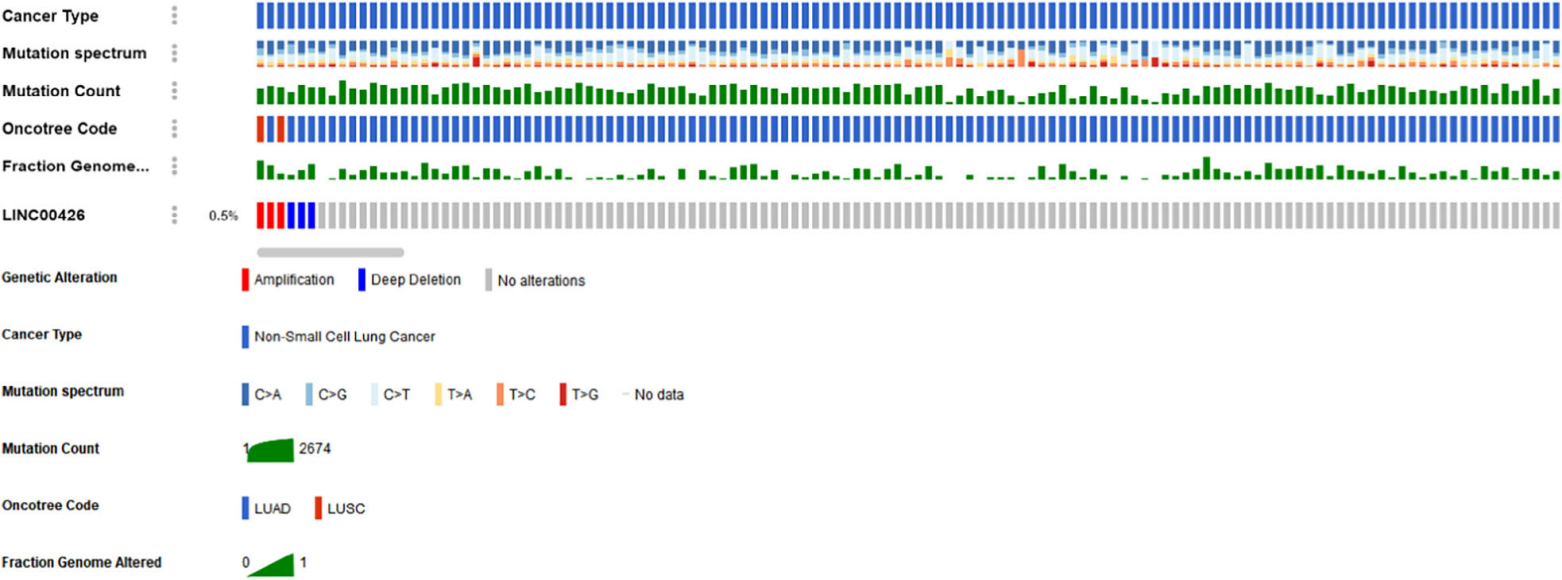
LINC00426 mutation analysis from the TCGA database.

The mutation of LINC00426 gene was analyzed from the TCGA database. Amplification and deep deletion mutation of LINC00426 gene was found in 0.5% of NSCLC patients (Fig 2).

### Survival analysis

**Figure 3:**
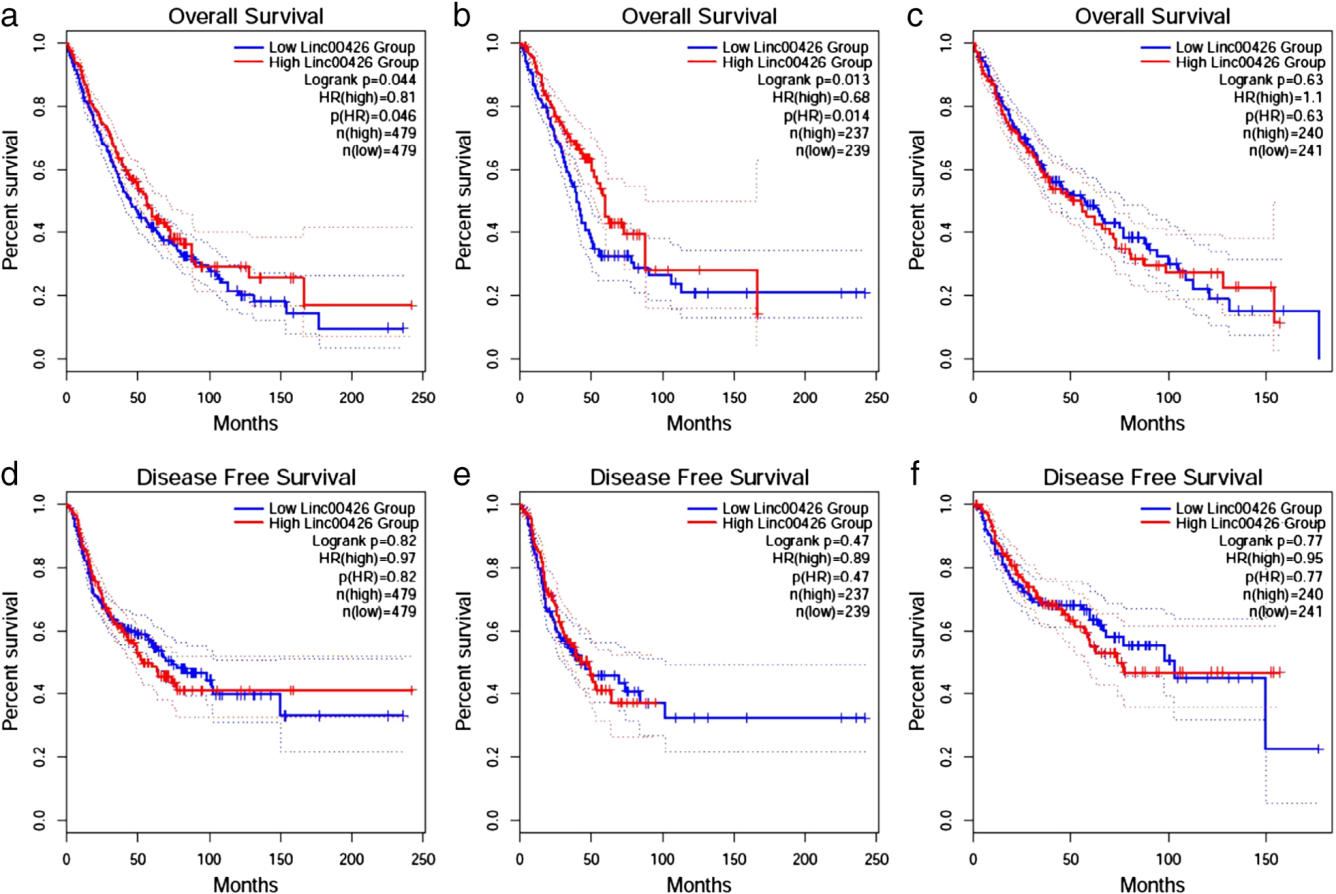
Correlation between LINC00426 expression and patients prognosis. (**a**) Overall survival for NSCLC. (**b**) Overall survival for adenocarcinoma of the lung; (**c**) Overall survival for lung squamous cell carcinoma. (**d**) Disease‐free survival for NSCLC; (**e**) Disease‐free survival for adenocarcinoma of the lung. (**f**) Disease‐free survival for lung squamous cell carcinoma.

According to LINC00426 expression level, the NSCLC patients were divided into LINC00426 high and low expression groups. Log‐rank test was used to evaluate the survival difference between both groups. The overall survival (OS) of LINC00426 high expression group was significantly higher than that of the low expression group (HR = 0.81, *P* = 0.044), while there was no significant difference between the high and low expression groups (HR = 0.97, *P* = 0.82) for disease‐free survival (DFS). Subgroup analysis showed that the overall survival of lung adenocarcinoma patients in the LINC00426 high expression group was significantly better than that of the low expression group (HR = 0.68, *P* = 0.014). However, LINC00426 expression was not correlated with the DFS and OS for lung squamous cell carcinoma (Fig 3).

### Correlation between LINC00426 expression and patients characteristics

**Figure 4:**
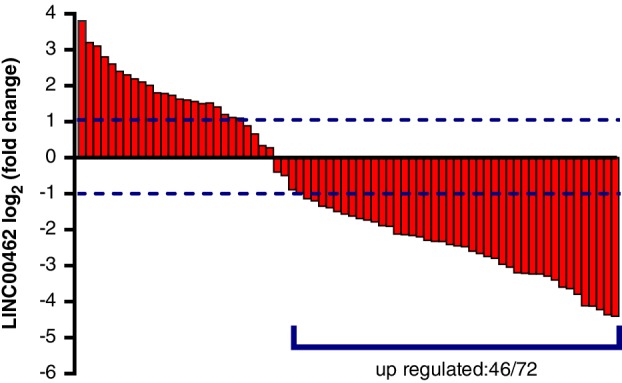
LINC00426 relative expression of the included 72 NSCLC patients.

LINC00426 expression was evaluated in 72 NSCLC patients. LINC0042646 expression level was elevated in 46 cases in normal lung tissue compared to the tumor tissue of the 72 NSCLC patients (Fig 4). LINC0042646 expression level was significantly correlated with the clinical stage of the patient (*P* < 0.05). However, LINC0042646 expression level was not correlated with age, gender, tumor diameter, pathology type and tumor differentiation (Table 1).

**Table 1:** Correlation between LINC00426 expression and patients characteristics

		LINC00426			
Characteristics	*n* = 72	High (*n* = 26)	Low (*n* = 46)	Chi‐square	*P*‐value
Gender				0.34	0.56
Male	42	14	28		
Female	30	12	18		
Age (year)				1.16	0.28
≤60	30	13	17		
>60	42	13	29		
Stage				2.01	0.04
I/II	50	22	28		
III	22	4	18		
Tumor diameter (cm)				1.72	0.19
≤5	56	18	38		
>5	16	8	8		
Pathology type				0.47	0.79
Adenocarcinoma	38	13	25		
Squamous cell carcinoma	30	12	18		
Others	4	1	3		
Differentiation				0.33	0.56
High/moderate	30	12	18		
Poor	42	14	28		

## Discussion

Long noncoding RNA (lncRNA) refers to noncoding RNA with more than 200 nucleotides.^8^, ^9^ According to the relationship between lncRNA and the location of protein coding genes, lncRNA can be divided into antisense lncRNA, bidirectional lncRNA, intergenic lncRNA and intragenic lncRNA. Previous studies have demonstrated that lncRNA can be detected in a variety of tumor and normal tissues.^10^, ^11^ lncRNA is involved in cells apoptosis,^12^, ^13^, ^14^ cells proliferation.^15^, ^16^, ^17^ It has been reported that long noncoding RNA Gas5 can regulate the binding of glucocorticoid receptors to response primitives, thus affecting the sensitivity of cells to apoptosis, and is therefore involved in regulating the invasion and metastasis of cancer cells.^18^, ^19^ Several studies have reported that lncRNA as a regulatory factor participates in the invasion and metastasis of cancer.^20^, ^21^, ^22^, ^23^ Long noncoding RNA HOX transcript antisense intergenic RNA (HOTAIR) can transinhibit the transcription of HOXD site by recruiting PRC2 complex acting as a scaffolding molecule.^24^ It can also recruit PRC2 complex and LSD1/CoREST/REST complex at the same time, resulting in different target gene‐specific histone modification changes, leading to cancer metastasis.

The expression level of lncRNA is significantly different in different tissues. Generally, the expression level of lncRNA is low in normal compared to tumor tissue. Some studies have shown that lncRAN had obvious tissue‐specific expression.^25^ Gibb^26^ systematically analyzed the data of differentially expressed lncRNA chips uploaded to the GEO database using bioinformatics technology. It was found that there were hundreds of differentially expressed lncRNA in tumor tissues and their corresponding normal tissues. Generally, most of the lncRNAs are highly expressed in tumors and low in normal tissues, suggesting that lncRNAs may play an important role in regulating the cancer occurrence, development, invasion and metastasis.

LncRNAs with high expression and known biological functions in lung cancer have been previously described and include MALAT1,^27^, ^28^ HOTAIR, Gas5, and H19^29^, ^30^, ^31^ Most of these lncRNAs are involved in the important biological regulation mechanism of the cancer occurrence, development, invasion and metastasis, and play an important regulatory role in lung cancer cells.

LINC00426 is a newly discovered long noncoding RNA located in chromosome 13: 30 340 267‐30 377 145 reverse strand with 12 transcripts in human beings. The biological function of LINC00426 is not as yet clear. In our study, we investigated LINC00426 expression in tumor tissue and corresponding normal lung tissue of NSCLC patients to determine its correlation with a patient's prognosis. We found that LINC00426 was downregulated in tumor tissue compared to normal lung tissue of the non‐small cell lung cancer patients and correlated with poor prognosis. Therefore, LINC00426 may be a potential biomarker for lung cancer prognosis. However, the biological function and relevant pathway of LINC00426 was not elucidated in our study and needs further investigation.

## Funding

This work was supported by Tianjin Health Bureau Science and Technology Fund Project (2014 KY26). Tianjin Science and technology program (18PTLCSY00080).

## Disclosure

None.
